# Supplementary material for: The Reduced Immunogenicity of Zoster Vaccines in CMV-Seropositive Older Adults Correlates with T Cell Imprinting
Source: Vaccines (Basel). 2025 Mar 22;13(4):340. doi: 10.3390/vaccines13040340 (PMC12031329; doi:10.3390/vaccines13040340)
Supplement: Supplementary file 1 [file vaccines-13-00340-s001.zip › vaccines-3469300-supplementary.pdf]

Table S1. Demographic Characteristics of Participants with Flow Data.

**Vaccine**		**RZV (N=30)**		**ZVL (N=30)**	
		**CMV-positive (N=19)**	**CMV-negative (N=11)**	**CMV-positive (N=21)**	**CMV-negative (N=9)**
**Median age** (IQR)		73 [64, 76]	70 [56, 74]	73 [59, 76]	71 [54, 75]
**Sex** N (%)	F	12 (63.2)	3 (27.3)	15 (71.4)	6 (66.7)
	M	7 (36.8)	8 (72.7)	6 (28.6)	3 (33.3)
**Race** N (%)	NW	2 (10.5)	0 (0.0)	1 (4.8)	1 (11.1)
	W	17 (89.5)	11 (100.0)	20 (95.2)	8 (88.9)
**Ethnicity** N (%)	NH	18 (94.7)	11 (100.0)	20 (95.2)	9 (100)
	H	1 (5.3)	0 (0.0)	1 (4.8)	0

Table S2. Antibodies used in the flow cytometry assays.

Marker	Format	Clone	Supplier
CD3	Ax700	UCHT1	Becton Dickinson
CD4	PC5.5	13B8.2	Beckman Coulter
CD45RO	PE-CF594	UCHL1	Becton Dickinson
CCR7	APC	3D12	Becton Dickinson
CD27	PE-Cy7	M-T271	Becton Dickinson
CD103	PE	Ber-ACT8	Biolegend
CD57	FITC	NK-1	Becton Dickinson
CD127	PE-CF594	HIL-7R-M21	Becton Dickinson
CD25	APC-Cy7	M-A251	Becton Dickinson
PD1	BV421	EH12.2H7	Biolegend
CLA	FITC	HECA-452	Becton Dickinson
LAG3	PE	3DS223H	eBioscience
TIM3	PE-CF594	7D3	Becton Dickinson
CD39	PE-Cy7	A1	Biolegend
CTLA4	APC	L3D10	Biolegend
CXCR3	APC-Cy7	G025H7	Biolegend
KLRG1	BV421	2F1/KLRG1	Biolegend
IL10	PE-Cy7	JES3-9D7	Biolegend
TGFb	APC	TW4-2F8	Biolegend
TNFa	APC-Cy7	MAb11	Biolegend
IFNγ	BV421	B27	Becton Dickinson
FoxP3	PE	259D/C7	Becton Dickinson

Figure S1. Gating strategy of T cell subsets pre-vaccination.

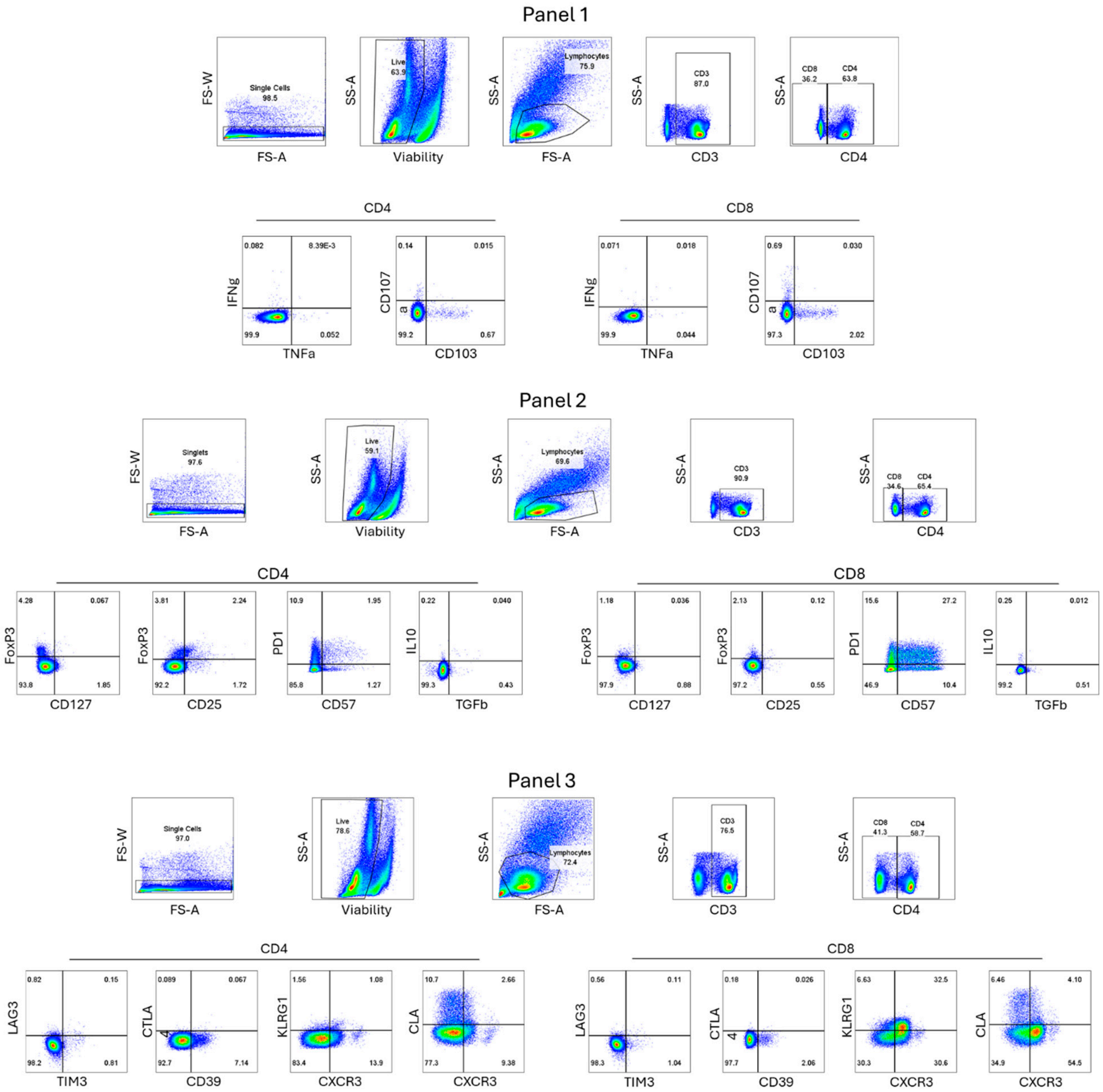

Figure S2: Consort flow diagram.

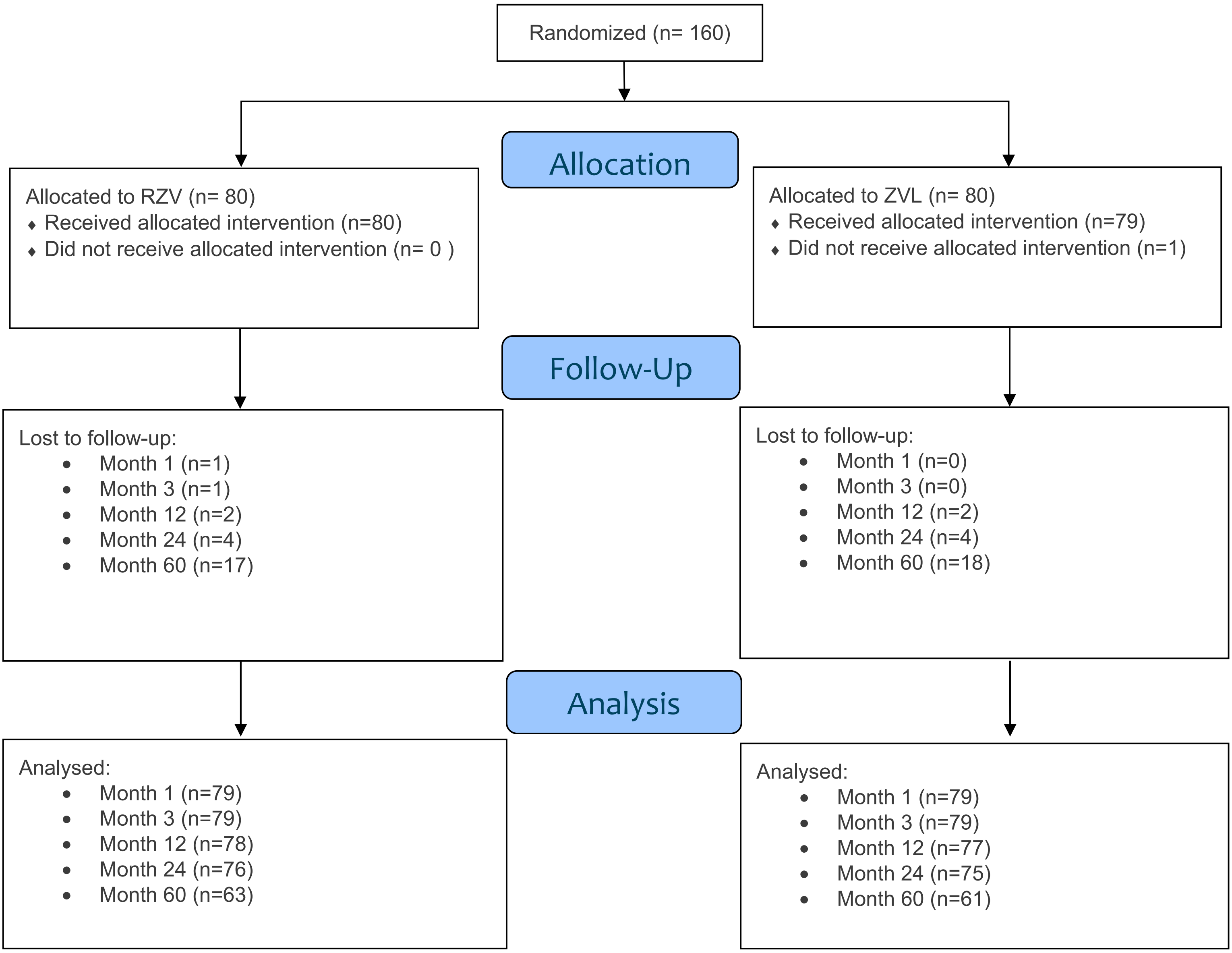

Table S3. Effect of sex on antibody avidity in ZVL recipients.

Months after vaccination	Effect size	P value
1	-5.65	0.19
3	-9.97	0.01
12	-0.84	0.79
24	-2.97	0.36
60	-1.87	0.62
